# Framing dementia experiences in a positive light: Conversational practices in one couple living with dementia

**DOI:** 10.1177/14713012211059028

**Published:** 2021-12-17

**Authors:** Elin Nilsson

**Affiliations:** Department of Sociology, Center for Social Work (CESAR), 8097 Uppsala University, Uppsala, Sweden; Department of Culture and Society (IKOS), Division of Social Work and Center for Dementia Research (CEDER), Linköping University, Linköping, Sweden

**Keywords:** dementia, couplehood, epistemics, frame analysis, conversation analysis

## Abstract

The general approach to a life with dementia is negatively charged, and alternative views are rarely found in research or in media coverage. This case-study explores conversational practices for framing dementia in a more positive light, employed by a husband of a wife with dementia. Framing regards the structured experiences of dementia, drawing on Goffman’s ‘Frame Analysis’. Benefitting from conversation analysis, this article presents principal results of four conversational practices used by the spouse without dementia: *mitigating trouble, normalising trouble, justifying trouble,* and *praising.* The conclusions drawn are that the practices contribute to the challenging of the dominant negative framework of the dementia experience, as they facilitate talk which emphasises the wife with dementia’s positive progression and skills in managing the household chores. Despite a positive framing of dementia, this couple still embed their talk in the overall negative framework of loss and decreased cognitive competence. The visualisation of a positive framing could add to a broadened view of dementia, which in turn could contribute to greater well-being for those affected. However, the results may also imply a risk of one spouse’s conversational practices of normalising and mitigating trouble being dominant in interaction and thereby neglecting the other spouse’s experience of the situation.

## Introduction

This study focuses on conversational practices employed by a husband of a wife with dementia for framing the dementia experience of everyday tasks in a positive light. The dementia (or major neurocognitive disability in DSM-V) umbrella includes a number of different diagnoses, Alzheimer’s disease (AD) being the most common one, followed by vascular dementia. AD is a degenerative illness which causes atrophy of brain cells, potentially leading to (among other symptoms) loss of memory and decreased ability to comprehend and to navigate in physical space as well as in conversation (Marcusson et al., 2011).

Receiving a chronic illness like a dementia diagnosis is often described as a dramatic turn of event in life leading to the experience of several present and potential future losses (Dupuis, 2002; Hellström & Taghizadeh Larsson, 2017; Moniz-Cook et al., 2006). The expectations of the future life with dementia are often negatively charged or framed (Behuniak, 2011). Goffman (1974) describes framing as the organisation of our experiences of a specific matter, shaped by as well as affecting our social world. Hence, the experiences of life with dementia are structured, understood and constructed within a specific frame, both by those with subjective and objective experiences of dementia. As spouses diagnosed with dementia have an increase in symptoms, couples may experience a negative change in marital quality and equality as spouses (Molyneaux et al., 2011). The approach to dementia may even affect the trajectory of the dementia in a positive or negative direction (Norton et al., 2009). So, how spouses within couples and families approach and talk about the dementia may have an impact on their experience of and management of everyday life over time (Roach et al., 2014) and may contribute to the general framing of the dementia experience.

This case study presents analysis of three interviews, conducted over a 3-year period, with one couple in which the wife had Alzheimer’s disease. The aim of this study is to identify and analyse conversational practices employed by the husband to create an alternative framing of the experience of dementia, one more positively charged than the mainstream negative frame. The study benefits from the methodological and theoretical framework of conversation analysis (Sidnell & Stivers, 2013),

### Dementia, couplehood and everyday experiences

A great deal of literature and media coverage is oriented towards the negative aspects of dementia, both in terms of present life as well as the negative aspects of the future. Overall, the social perception of the trajectory of dementia is characterised as ‘going downhill’ (Ashworth, 2020; Behuniak, 2011; Harvey & Brooks, 2019; O’Connor et al., 2018; Peel, 2014). Despite the dominant negative framing of the dementia experience and everyday life, research in the forefront rather puts forward a perspective which emphasises remaining strengths and abilities despite dementia (Hydén & Antelius, 2017). McGovern (2011: 678) introduces a strengths-based approach with ‘the possibility of a positive outcome’ despite dementia, one that is rooted in relationships and strengths. From this perspective, togetherness rather than separateness is central, and spousal couplehood is emphasised as an important component for achieving well-being (Bielsten et al., 2018; McGovern, 2011; Swall et al., 2020). The couplehood perspective to ‘diminished everyday competence’ for a spouse diagnosed with dementia has also been singled out as important for a continued sense of agency in daily activities (Hellström & Taghizadeh Larsson, 2017; Hellström & Torres, 2021). Couples living with dementia jointly frame their experience by co-creating, defining and assessing the quality of their daily life in their shared talk and adjustments in regard to their situation (Bielsten et al., 2018; Hellström & Taghizadeh Larsson, 2017). By doing so, they will most probably also influence their own experience of their current situation, regardless of whether the framing is positively or negatively charged. This strength-based perspective also increases in representation in the media coverage of dementia, where agency and the ability to ‘do something’ to live well with dementia receives more attention (Peel, 2014).

Several research studies (e.g. Gallagher & Beard, 2020; Hellström & Torres, 2016; Hellström & Torres, 2021; McCabe et al., 2018; Nilsson & Olaison, 2019; Swall et al., 2020), have described how couples and families living with dementia may adopt the approach of ‘taking it day by day’ rather than worrying about the future and thereby enabling momentary well-being despite a negative trajectory of the dementia. Caring spouses put great effort into sustaining the sense of self-esteem and agency for a partner with dementia, and maintaining daily activities are central (Hellström & Taghizadeh Larsson, 2017). Hellström and Torres (2021) describe three different approaches that spouses of a person with dementia may take in regard to manage their spouse’s ‘diminished everyday competence’ (DEC) – as in a weakened ability to manage daily chores. The first approach is that the spouse of a person with dementia ‘diminishes the impact’ the DEC may have on their daily life. The second is that spouses talk from an egocentric perspective and ‘stress the impact the DEC has on their own daily life’ rather than the life of the couple. This approach would put the person with dementia in a vulnerable position of being a burden to their spouse. The third approach is to ‘emphasise couplehood’ as a departure for experiencing DEC. Hellström and Torres (2021) also argue that in their data these patterns are valid regardless of the level of the dementia progression. On a similar note, Foster (2011) states that practices employed by couples living with dementia involves restoring normality through means such as meaningful activity and staying active, but also by straightforward discussions and formal support (Foster, 2011).

### Dementia and conversational collaboration

Talking and telling stories have been described as performative activities, in which participants may display relationships (Goffman, 1971; Mandelbaum, 1987), identity (Hydén & Örulv, 2009) or other aspects of life. As dementia affects cognition and executive functions, persons with dementia also often have difficulties with word-finding, orientation and keeping track in conversation, which provide challenges in social interaction (Marcusson et al., 2011). Encounters involving couples living with dementia also often entail descriptions of all the things that the person with dementia can no longer do or remember (see Nilsson, 2018). These descriptions may put the person with dementia in a vulnerable position where s/he is described in terms of shortcomings rather than strengths, contributing to an overall decrease in well-being and low status (McGovern, 2011). Within families and couples, talking of how life is, and how it has changed and will change due to the dementia can therefore be described as a sensitive matter which requires careful attention and further knowledge (e.g. Landmark et al., 2021).

A conversational partner such as a spouse may increase the abilities of the person with dementia to participate socially and tell stories in a competent manner, through for instance narrative scaffolding and repair sequences (Hydén, 2018; Kindell et al., 2016; McCabe et al., 2018) but also through mitigated corrections (Landmark et al., 2021). Hydén (2018) argues that the telling of a story when having dementia may be more of a collaborative and interactive project than for people who do not have this condition*.* Telling a joint story can also be a way for couples to present themselves as a ‘we’ to the world, by marking and emphasising togetherness in their storytelling (Goffman, 1971; Mandelbaum, 1987). The shared storytelling may also well be a way to retain a prior relationship, as couples’ storytelling often draws on joint remembering and co-telling of shared experiences that both have unique access to (Hydén, 2018).

### Dementia and epistemics

In conversations, there are social rules regarding who ‘ought to know what’, and who ‘has the right to talk about it’ to others. In conversation analytical research, these rules are often understood in terms of ‘epistemics’, which is a concept that concerns the ‘knowledge claims that interactants assert, contest, and defend in and through turns-at-talk and sequences of interaction’ (Heritage, 2013: 370)*.* Normally, we ‘own our experiences’ in the sense that the person who has first-hand knowledge of something such as an event, also has the primary rights and obligations to formulate this experience to others as well as to make assessments of it. This has been referred to as having different ‘epistemic territories’ (Heritage, 2012). The violation of the social epistemic order has been referred to as ‘epistemic trespassing’ (Bristol & Rossano, 2020), where one person takes the liberty of talking about something which belongs to another person’s domain. However, in close relationships there may be more allowance for this as the epistemic boundaries are not clear-cut. Within interactions involving persons with dementia, this order may be even less strict. Dementia can be regarded an ‘epistemic impairment’ (Bristol & Rossano, 2020), as it comes with decreased authority over core epistemic domains.

In talk of daily matters or knowledge regarding dementia, both spouses in a couple may have equal rights to the talking about something which only one of them actually has experienced (Landmark et al., 2021). Previous research drawing on audio-recordings has emphasised that the voices of persons with dementia are often neglected (Österholm & Samuelsson, 2015). However, when analysing also visual data, it was found that persons with dementia may recurrently hand over speakership of first-hand knowledge via gaze or verbal conduct to their spouse, displaying a shift in epistemic rights and primacy to talk about first-hand knowledge. For a couple living with dementia, this knowledge may even concern the person with dementia’s physical and psychological state due to dementia (Nilsson et al., 2018). For persons with dementia, the issue of not remembering, or accessing first-hand knowledge is recurrent in social encounters. Svennevig and Landmark (2019) present different conversational practices for accounting for lack of knowledge or memory in interaction involving persons with dementia. These practices are to ‘normalise’ the lack of knowledge by treating it like something that could happen to anyone, to ‘exceptionalise’ it by marking forgetfulness as deviant from a person’s normal cognitive state, and to ‘justify’ the matter by treating it as not important and/or reasonable to forget (Svennevig & Landmark, 2019).

Although the general framing regarding dementia is characterised by negative stereotypes, losses and dark prospects for the future a great deal of current literature emphasises the possibilities embedded in collaborative storytelling and conversation in terms of abilities for persons with dementia. In other words, there are tendencies for the inclusion of a more positive framework, an alternative which requires analysis on a micro-analytical level (Goffman, 1974: 25). This positive framing can be enabled also by couples living with dementia. Previous research has found approaches employed in daily life to manage the symptoms of dementia, such as diminishing its impact on their life, but also emphasising its impact on the individual or the *couplehood* (Hellström & Torres, 2021). The more interactionally oriented research presents conversational practices with the function to account for the forgetfulness, by normalising, exceptionalising or justifying it (Svennevig & Landmark, 2019). The current study adds to this research by identifying and analysing conversational practices employed by a spouse in one couple when ‘framing dementia in a positive light’, talking specifically about the daily challenges and progression. To explore and identify the components of the talk may be important to help couples gain an experience of greater well-being and a positive outlook while still living with dementia. Moreover, remaining strengths and abilities despite dementia may surface. This study contrasts with most previous empirical research as this couple frames their story differently to the general discourse on dementia, when referring to their current situation as improving rather than declining over time.

## Method

### Interviews

The analysis for this study draws on three video-recorded interviews over a three-year period (totalling 1.52 min) with one married couple. The overall material consists of approximately 30 h of video-recordings of 52 interviews during 5 years with the same 15 couples. The interviews were conducted by researchers at Centre for Dementia Research (CEDER) between the years 2011 and 2016. All participating couples were assigned narrative tasks to talk about (becoming a couple; dementia, everyday chores, etc.); new tasks were added at each interview. This design aimed at eliciting conversational collaboration between the spouses. During the interviews, couples were given plenty of time to jointly tell and pursue their stories. The participants were seated in an F-formation around a small table; all were facing each other and separated with a small table (Kendon, 1990). The same interviewers, one male professor in social psychology and one female associate professor in nursing, were present in all three interviews. The spouses’ talk within this multi-party interaction can be addressed to the other spouse or addressed to the interviewers. As the talk was hearable for all participants, the descriptions of the couple’s life that the spouses shared in the interviews ought to be understood in terms of constructions potentially aimed at a third-party (the interviewers) rather than ‘correct images of their life’.

### Participants

The interview participants for this study were the married spouses ‘Kari’ and ‘Mathias’ (fictive names, K and M) and the two researchers who interviewed (I1 and I2). The couple had been married for 43 years at the first interview, and they had two grown-up children. They were living at home with no formal support. The wife, Kari, was recently diagnosed with Alzheimer’s disease at the time of the first interview. In the first and second interviews the couple described how Kari had received some ‘special medication’; however, we as researchers had no additional information about the type of treatment or the results in terms of dementia symptoms. Also, there is no information regarding scores on FAST (Reisberg Functional Assessment STaging scale) or MMSE (Mini-Mental State Exam); however, the couple described mild symptoms regarding executive functioning but stated that Kari’s main problem was related to word-finding.

### Analysis

The analysis is divided into conversational practices employed by the husband of a woman with dementia. In contrast to findings from previous research, this couple’s talk regarding the wife with dementia’s development and abilities is more positively framed. The selected cases from all three interviews for this study are all about the couple’s descriptions of practices regarding the wife’s development and management of daily household chores (i.e. baking, cooking, sewing, doing laundry and dishes). In the analysis of the spouses’ talk, several conversational practices used by the husband to facilitate talking about dementia-related matters were identified.

The study benefits from the methodological and theoretical framework of conversation analysis, which draws on sequential analysis of the participants’ understanding of the situation. Conversation analytical data consists of video or audio recorded interactions. These are transcribed, including details regarding for instance prosody, pauses, overlaps and non-verbal contributions such as gaze and gestures for a richer image of ‘what is going on’ in the interaction. In conversation analysis, each interaction is analysed individually in terms of sequential organisation, and may thereafter be connected to other similar cases and make a collection of a phenomenon (Schegloff, 1987; Sidnell & Stivers, 2013). In more atypical interaction such as involving a person with dementia, there is an increasing interest in case studies (single or collective cases) (see Schegloff, 1987) as well as longitudinal analysis in order to capture progression and changes (see Hamilton, 1994; Kindell et al., 2019). In this study, conversational practices employed by one spouse is in focus; hence, the interaction between the spouses receives limited space in this analysis and is mainly emphasised in terms of possibilities for collaboration and joint storytelling.

The concept of ‘social framing’ (Goffman, 1974) is used to provide an overall description and understanding of the participant’s talk in regard to the matter. Framing concerns the organisation of experiences of a specific matter, shaped by and affecting our social world. However, in regard to normative frameworks, such as that of the dementia experience, Goffman (1974: 346–347) states that ‘…some deviation is tolerated. And if effective cover is maintained, a great deal of deviation can be got away with’. In this current study, the couple deviates from the normative social framing of dementia as ‘going downhill’ by co-constructing an alternative dominant framework for their own situation which is more positively charged.

The current analysis also benefits from the theoretical concepts of ‘epistemic territories’ which has been described (e.g. Heritage, 2012) as well as ‘politeness theories’, which regards the upholding of a positive face for oneself and the moral obligation to support each other’s face in interaction (Goffman, 1967; Brown & Levinson, 1978). In the following, conversational practices used by the husband to facilitate a positive framing of the dementia experience will be presented and analysed.

## Results

The analysis is presented as four different conversational practices employed by the spouse without dementia for framing dementia in a positive light. The practices are *mitigating trouble,* as in presenting the trouble as having low impact of their life*, normalising trouble,* as in suggesting that symptoms are possible for all people, *justifying trouble,* as in providing an account which explains the trouble and *praising,* as in the spouse praising the abilities and performances by the wife with dementia. In total, these practices were found at 32 occasions over the three interviews, but as the practices overlap occasionally the figures are not presented individually. When conducting longitudinal comparison, no specific change can be seen in terms of the practices employed. However the results indicate that the framing of dementia in a positive light is strengthened over time. The wife with dementia is initially more hesitant in her positive assessments of their situation but in interview two and three she provides a more elaborated positive framing in co-construction with her husband.

The wife described her main trouble to be that she ‘loses words’, something that was vivid throughout all interviews with this couple. The most common supporting practice overall was therefore that her husband provided the words his wife was missing, mostly solicited by an invitation from her to come in and support her. However, this practice will not be presented in this article, as it was not specifically tied to the creation of a positive framing of a dementia experience. It has been described in previous work by the research group (see Hydén, 2018; Hydén & Nilsson, 2015).

The English translation is presented in bold and italics. When relevant, the non-verbal details are included within double brackets or with aligned marking; see transcription key in the appendix, following conventions by Jefferson (Jefferson, 2004) with non-verbal additions. Laughs or minimal contributions are included in English only.

### Mitigating trouble

These first examples (divided into Ex.#1a and Ex.#1b) are drawn from the first year’s interview with the couple. The extract is a continuation of Mathias’s response to a question posed by the interviewer which concerned whether he notices the word-finding difficulties, previously described by Kari who has dementia. Just prior to the Ex.#1a Mathias responds that he does notice these difficulties, and that he does not find her problems to be getting better. However, it becomes evident in the continuation here that he does not regard them as getting worse either, and he downplays the impact the troubles have on the couple’s daily life, an approach also found by Hellström and Torres (2021).

The sequence starts when the husband Mathias (M) states that he personally does not think his wife Kari’s (K) symptoms of dementia have become worse; rather on the contrary, they have stabilised somewhat (line 1). Ex.#1a not only shows how the couple describe what they feel about their daily life but also shows how they manage talking about it.


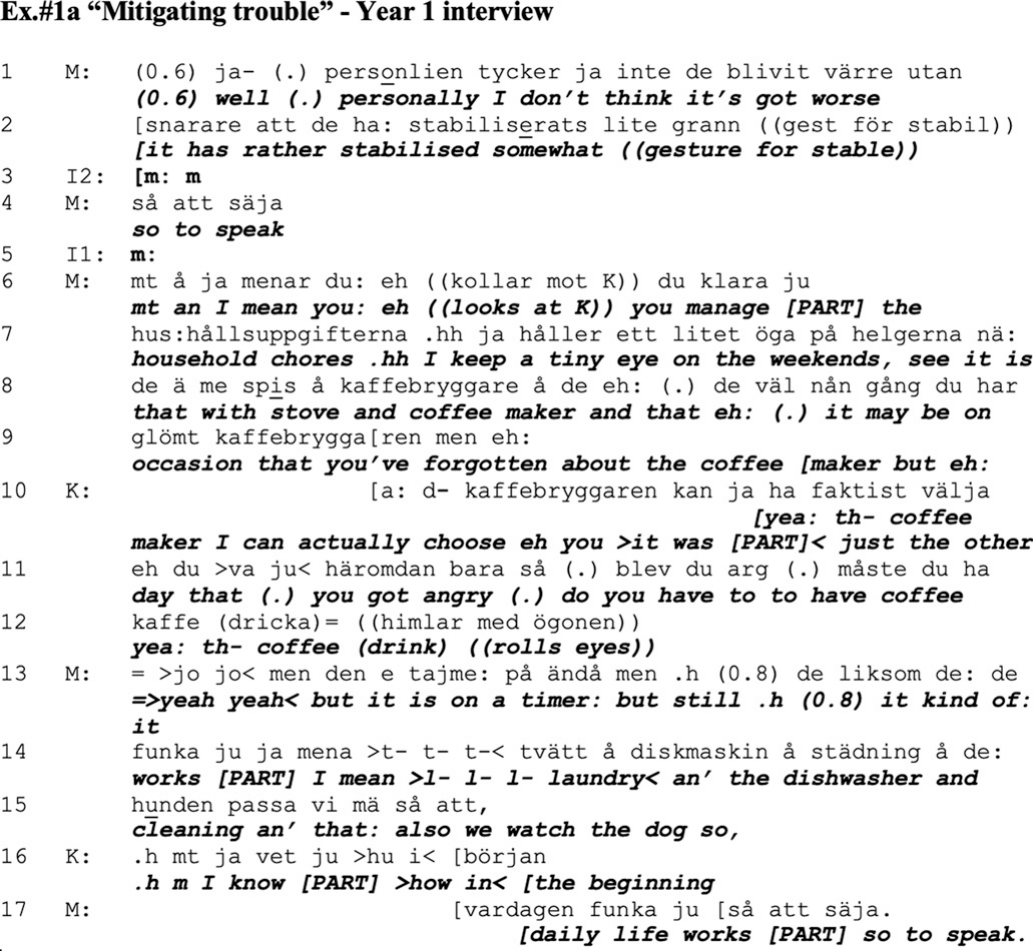


In this sequence, Mathias actualises the taken for granted knowledge in the talk, namely, that the trajectory ought to be going downhill due to the dementia. He does this by marking an opposition in that he ‘personally doesn’t think it has gotten worse; rather it has stabilised somewhat’ (line 1). He then turns to his wife Kari and lists all the things she manages to do; however, with negative assessments displayed in mitigating words such as ‘keep a tiny eye’ and ‘it may be on occasion’ (lines 7–9). So, Mathias’ assessment of Kari’s management ‘you manage [PART ‘ju’^1^] the household chores’ (lines 6–7) is followed by evidence for this assessment but also concessions, which partly moderates his initial approach (Lindström & London, 2014) (lines 6–9). These negative assessments are also instantly followed by a ‘but’ (lines 9 and 13) which could indicate that these should be understood as exceptions to the assessment and framing of the experience in a positive light, similarly to the practice of ‘exceptionalising’ in Svennevig and Landmark (2019). In his talk, Mathias describes how he keeps an eye on his wife when she is doing chores, and thereby indicates that he is superior in terms of cognitive capability. Here, Kari interrupts and affiliates with her husband’s assessments regarding her occasional difficulties, while also adding a description of when she had a problem regarding the coffee maker, partly in jokingly reported speech of her husband ‘do you have to have coffee’ (lines 10–12).

Although Mathias affiliates with Kari’s description and assessment of her troubles, he yet again returns to his previous track which was to describe all the things that she manages to do (lines 13–15). Kari agrees but also elaborates by attempting to provide a chronological take on her symptoms (line 16); however, she is interrupted by her husband who concludes by saying that ‘daily life works alright [PART] so to speak’ (line 17). Kari is initiating a historical perspective with details regarding how she used to be, which overlaps with Mathias’ summing up of what has been said (line 17).

In this sequence, both spouses appear to co-construct a telling, which aims at framing the image of daily life as going alright, although with some exceptions (i.e. Svennevig & Landmark, 2019). In their talk, they imply that Mathias has superior epistemic status (Heritage, 2012) regarding the household and security, embedded in descriptions regarding the managing of the coffee-maker and stove, where Mathias ‘keeps an eye on her’. Both Kari and Mathias describe occasions when Kari failed to manage, but Mathias balances these descriptions with elaborations on all the things she *can manage*, and mitigates the effect that these lapses have on their daily life (i.e. Hellström & Torres, 2021). Initially, as Mathias is more active in the conversation, he appears more engaged with the positive framing. Potentially, he even overrides his wife’s assessments of their situation as will become vivid in this following example (Ex.#1b), which follows Ex.#1a although omitting a few lines in between. The focus here is Kari’s own assessments rather than Mathias’s, where she provides a detailed historical background of her challenges and the strategies employed for coping with dementia symptoms. Mathias yet again provides mitigations of the problems described by Kari.


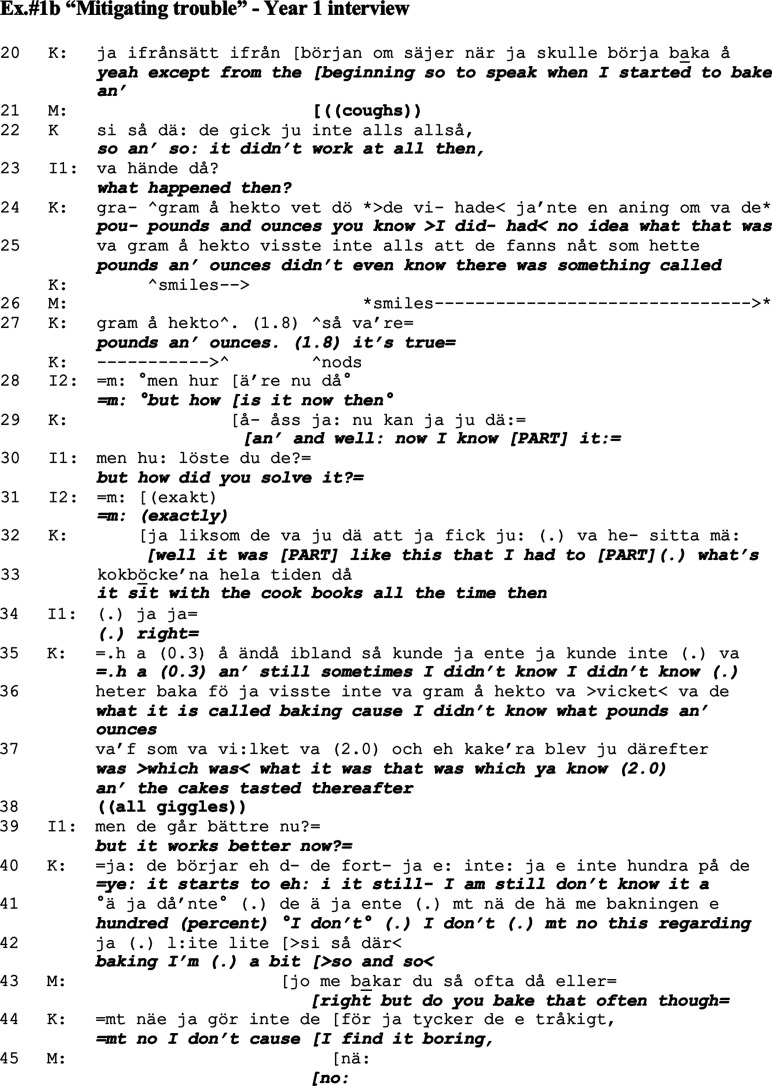


Kari provides a background to the current situation which displays a development of moving from worse to better in terms of her ability to cope with daily chores, and she provides detailed descriptions of the procedure of looking closely at cook books, and returns to her issue of not knowing about pounds and ounces.^2^ Throughout her talk, Kari skilfully provides relevant resources regarding the time line, ‘in the beginning’, ‘had no idea’, and ‘now I know how to do it’, and through that comes across as competent in speaking about a relevant topic. In her talk, Kari also displays competence as she explicates that she now knows something specific, about pounds and ounces, something that she says she previously (early in the dementia trajectory) did not know even existed. She also manages to competently display that she now knows how to use something which she previously did not even know existed as a concept. When finished, Kari ‘returns home’ (Mandelbaum, 1987) with a humorous ending regarding the quality of the cookies at this time (line 37), a joke that receives giggles from everyone.

The interviewer then returns to the present narrative with a confirmation-seeking statement, thereby participating in the co-construction of the same narrative line of things working better now (line 39). Kari agrees moderately with the statement, but provides a more diversified image of how she still experiences some issues when baking (lines 40–42). Kari’s turn indicate that she is not as engaged to the positive framing as Mathias, who joins in and downplays the importance of Kari’s description of her problems through mitigating talk as ‘Well but do you bake that often though?’ (line 43). The downplay by Mathias justifies Kari’s troubles with baking since when presenting it as a rarity (i.e. Svennevig & Landmark, 2019). Kari affiliates with Mathias by responding that she does not bake much since she finds it boring. Here, Kari’s extended descriptions of trouble get embedded in a mitigation as well as a justification of her trouble, and thereby the positive framing gets precedence over the negative in the end.

By asking Kari if she actually does bake that often nowadays, Mathias also enters the epistemic domain of his wife as baking is (or was) her hobby (see Heritage, 2012), although in a tentative manner. His question implies a decreased impact of the trouble described by Kari on their daily life, similarly to the pattern of ‘disregarding diminished competence’ (Hellström & Torres, 2021). In the sequence of talk in Ex.#1a and Ex.#1b Kari attempts to describe difficulties related to her dementia as an opposing view to that expressed by Mathias, knowledge which is within her own domain. Although Kari has the primary right to talk about this knowledge (Heritage, 2012), Mathias enters her domain by questioning the relevance of her described experiences of trouble. This entrance would fall under the category of ‘epistemic trespassing’ (Bristol & Rossano, 2020); however, it could also be seen as a supporting practice which opens up for a more positively framed talk of his wife’s difficulties.

In relationships, this type of epistemic trespassing may even display a couple’s relationship as intimate rather than general (Bristol & Rossano, 2020; Landmark et al., 2021). Kari agrees with Mathias’s questioning of her baking habits, and also adds a ‘justifying account’ for this (Svennevig & Landmark, 2019), ‘because I find it boring’ (line 44). By doing so, Kari indicates that this mitigation and entering of her epistemic domain was acceptable, as she continues by adding to this topic further in the direction proposed by Mathias. In this turn, Mathias also shows that he is a competent as well as an accepted co-teller of the story initiated by Kari, as he provides a relevant summon, or mitigation of trouble, which facilitates further talk by his wife. Mathias’s contribution here (line 43) also opens a possibility for Kari to preserve a ‘positive face’ (Brown & Levinson, 1987) in spite of troubles when baking, by describing the minimal occurrence of such trouble as well as providing an opportunity for Kari to account for it herself as it is stated as a question. In that sense, the agency and epistemic order of Kari knowing best what she can do are preserved (Heritage, 2012).

### Normalising trouble

The normalisation of dementia related symptoms is a common approach of resilience or coping when living with dementia (i.e. Foster, 2011; Svennevig & Landmark, 2019). In the midst of the second interview one year later, the couple yet again talk about baking and Kari’s ability to manage daily chores. The interviewer returns to a topic previously initiated by Kari by asking about potential strategies Kari has used in order to resolve her issues with not being able to do the baking, or if her success was driven by her described anger and feelings of revenge towards the geriatric clinic. In Ex.#2, Mathias adopts the practice of normalising Kari’s symptoms by means of self-deprecation and claiming that baking is difficult even for him, thereby supporting the positive assessments of Kari’s abilities. By making it evident how difficult he himself finds baking and the cooking measurements, Mathias forefronts how difficult the measurements actually are since he is a cognitively intact person compared to Kari, and thereby normalises her troubles and supports the positive framing.


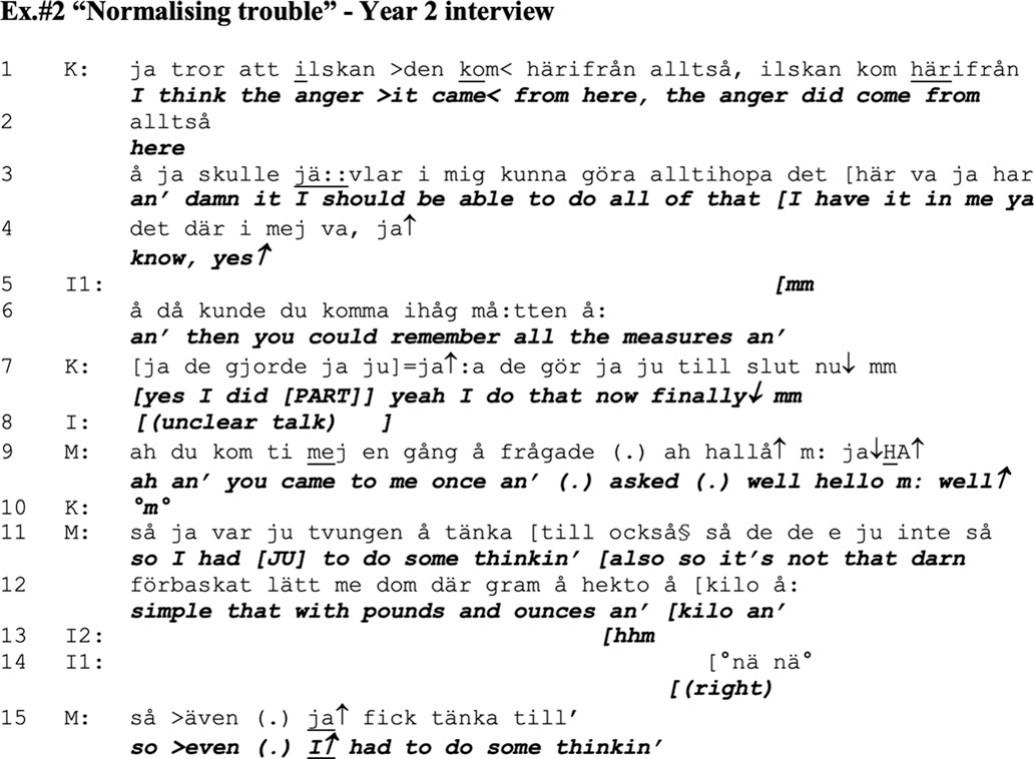


Kari argues that it was her anger towards the geriatric clinic that enabled her to manage the measurements when baking ‘[swearing/damn it] I should be able to do all of that, I have it in me’ (lines 3–4). This is responded to by the interviewer with the confirmation-seeking statement ‘and then you could remember all the measures an’ (line 6), a statement which Kari affiliates with by declaring that she managed and now she finally knows the measurements (line 7). At this point Mathias joins in and tells of one time when Kari asked him for help, and he found himself unable to help immediately and he had to think as (in joking tone) ‘it is not so darn simple with those pounds and ounces an’ kilo an so even I↑ had to think’ (lines 11–12, 15). Through Mathias’ self-deprecation he normalises Kari’s troubles, and she comes across in a more favourable light as the task is described as difficult even for a cognitively healthy person.

In this excerpt, Kari is actively contributing to the positive framing by indicating that her ability to manage baking and cooking measurements comes from her ability to ‘fight through’ troubles (line 3), not connecting it to the medication which she and Mathias had told the interviewers about previously. Mathias supports this project of making positive assessments of Kari’s skills by providing an example of when he himself was not able to work out the cooking measurements, indicating that the thing his wife now is able to do is actually quite difficult for anyone (lines 9–15). In this contribution, Mathias uses resources such as emphasis (‘came to me’) and verbal design (‘so I had [JU] to do some thinking’) to show what is often taken for granted in interaction involving a person with dementia, which is the asymmetry regarding competences between persons with or without dementia. By emphasising that even *he* had to think about the cooking measurements, he indicates in the same manner as in Ex.#1a that he is expected to know this, in contrast to his wife with dementia.

Mathias’s contribution (lines 9–15), does not only show and establish the landscape of who can do what, it also works as an encouragement for Kari to continue to talk (below in Ex.#3, lines 16–23). Mathias’s formulation even opens a possibility for Kari to initiate the potentially risky business of self-praising. As Mathias states that *not even he* can manage easily, Kari can pursue this positive framing by elaborating on the details and ‘proof’ of how she is coping more than well or as well as anyone. This type of normalisation of dementia-related troubles has also been reported by Svennevig and Landmark (2019); however, with the focus on not remembering an occasion or a word, rather than not being able to perform a practical daily task, which is the issue in Ex.#2. Thereby, the two studies connect by the placing of dementia in the background and the ‘normal’ at the front.

### Justifying trouble

In this continued talk which follows the previous example of normalisation, Kari herself provides several detailed evidential stories about her managing cooking for the more special occasions such as Christmas and birthday parties which require more work and cognitive effort. However, Kari has trouble finding the main word in this description (rice), so when Mathias enters as a speaker and provides the correct word (line 22) he also provides a detailed justifying account of the circumstances which caused his wife’s trouble in regard to prepare the rice (i.e. Svennevig & Landmark, 2019).


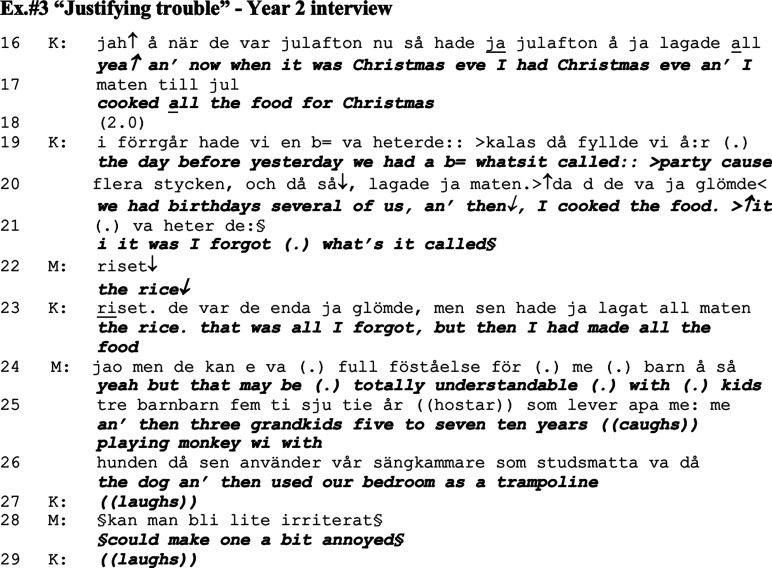


In this sequence, Kari formulates her accomplishment with self-praise, great detail and emphasis with words like ‘I cooked all the food’. As the path for self-praising is already paved (which we saw in Ex.#2), Kari’s self-praise can be expressed with no accounts or excuses from her end. When she tells of the tiny problem she had with the meal, namely forgetting the rice, Kari has trouble in talking about this as she forgets the word ‘rice’ (lines 20–21), but the word is then instantly provided by Mathias (line 22). After Kari’s affirmation that this was the word that she was searching for, she returns to her story by also using the mitigating words ‘the only’ (thing I forgot) in relation to the rice incident and repeating that she herself ‘made all the food’ (line 23). Mathias then provides a justifying account for why it would be easy to forget the rice, as there were children, several younger grandchildren and a dog that were all playing in their bedroom (lines 24–26, 28). This utterance receives laughter from Kari and finalises the story she had initiated. Yet again, Mathias’ contribution of providing an account here can be seen as a way of contributing to and ratifying his wife’s self-praise in regard to managing her chores despite occasional shortcomings. Mathias’ detailed account provides evidence for a justification for Kari’s forgetfulness, which Svennevig and Landmark (2019) also found in regard to forgetfulness during interaction. Overall, the practice of normalising and justifying trouble is responded to by Kari with self-praise, and it opens a possibility for a strengthened positive framing of her abilities.

### Praising

In the midst of the third and last interview, Kari yet again returns to the topic of the indoor daily chores that she manages well. In the interview, prior to the excerpt in Ex.#4, Kari has described how she can easily manage doing the laundry and wraps up this talk by assessing their situation in a positive light (line 1), which is where this excerpt below begins. This assessment added on and upgraded by Mathias in terms of giving credit for her abilities, which in turn elicits further self-praise for Kari. At this point, she also invites her husband to speakership by looking at him (line 1) and pauses (line 5), potentially to confirm her contribution. In similar to previous example, Mathias self-deprecates by indicating that he himself is redundant in terms of indoor chores (line 7), and he also provides praise of all the things his wife does around the house.


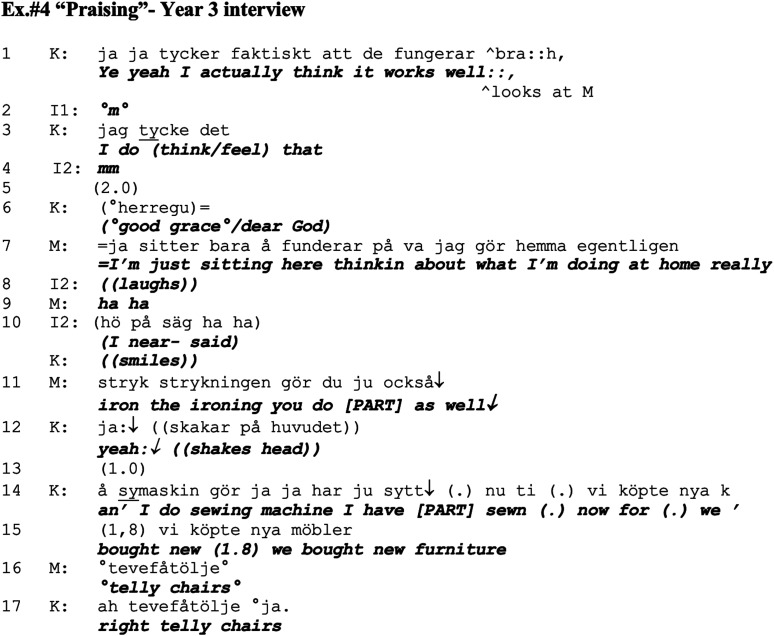


Kari states that she thinks it (presumably daily life/dementia symptoms) is going well (line 1). Kari uses the more imprecise noun ‘it (works well)’ rather than ascribing herself this praise (as in ‘I’m (doing well)’), which could also possibly explain the lack of account from her end for this positive assessment. The word ‘actually’ (line 1), indicates that this ought to be understood as something surprising or extraordinary rather than expected (Wilkinson & Kitzinger, 2006), which fits with a general expectation of failing due to dementia. There is a preference displayed in Kari’s contributions (lines 1, 3), which is to include Mathias in the positive assessment via her gaze at Mathias and also by repeating her subjective assessment. However, Kari’s talk is not responded to by Mathias, instead Kari receives minimal continuers by both interviewers. After the two-second silence (line 5), Kari adds the post-completion stance marker ‘good grace’ which may be interpreted as a way of stating that this is the opposite of the expected trajectory of life with progressing dementia. This may be an interactively troublesome situation for Kari as the positive aspects that she describes relate to herself and her ability to manage daily chores; hence, she is both the subject of making the assessment as well as the object that is being assessed. This situation has been considered questionable in terms of believability as well in regard to having the epistemic primacy of making such assessment, that is, it would be preferable if the praise came from a third party in direct or reported speech (Pomerantz, 1984; Wilkinson & Kitzinger, 2006). This could be one of the reasons why she turns to her husband, to enable ‘epistemic distance’ to the matter from a third party (Wilkinson & Kitzinger, 2006) and thereby also save ‘positive face’ (Brown & Levinson, 1978; Goffman, 1967).

Although Kari turns to Mathias by moving her head and gaze in line 1, he does not respond to this until line 7, after several turns by his wife, a long pause, and the expression ‘good grace’. When Mathias joins in by adding to Kari’s evaluation of how well things are going, he does this with the upgrading formulation in which he credits Kari’s performances at home by downplaying his own ‘I am just sitting here thinking about what I’m doing at home really’ (line 7). This self-deprecation is something that is clearly understood as humorous as it receives laughter from the interviewer and smiles from Kari. The use of humour to show the gendered division of household chores has previously been described by in the work by Flinkfeldt (2014) in relation to online chats and counterbalancing risks of being seen as a ‘housewife’ when doing household chores. In his formulation, Mathias also accounts for his passivity during his wife’s talk by saying what he was thinking about during that time and thereby he also counterbalances the potential display of disalignment to the communicative project, which is often connected to this type of silence (Lee, 2013). Furthermore, Mathias provides evidence of his statement in that Kari also does the ironing (line 11). Kari then takes over and adds to the husband’s praising by saying how she also manages the sewing machine, and she also describes her latest work on this machine in detail. Kari wraps up this talk with a humorous description of her sewing work (not included here), yet again displaying skills in terms of balancing the describing of details in a ‘storytelling manner’ (Mandelbaum, 2013).

By downgrading his own participation in the household chores, Mathias also acts as a third-party praising Kari and ratifying her earlier self-praise (Speer, 2011). It is especially significant that it is Mathias who provides this rather than someone else, as he is an expert witness being both Kari’s spouse as well as present on the occasion described. Speer (2011) describes a similar situation in which transgender persons report praise given by their family members regarding their ‘coming across as transgender’ in a good way, and how this is used as a way of ratifying further self-praise on the topic. Similar to the patterns in previous examples, Mathias contributes in a way that makes it possible for Kari to ‘shine’ and self-praise without losing face (Brown & Levinson, 1978; Goffman, 1967). However in Ex.#4 Mathias’ contribution is with maximal upgrade as he explains that he cannot even think of anything that he does around the house in terms of household chores.

## Concluding discussion

The analysis has identified several conversational practices used by a husband without dementia to enable a ‘positive framing’ in talk of dementia-related experiences, as well as to include his wife with dementia in the talk. The practices which have been described are *mitigating trouble, normalising trouble, justifying trouble* and *praising.* The analysis shows how the spouses balance positive and negative assessments of the wife’s past and current ability to manage household chores despite dementia and suggests that a slight deviation from the negative framing of dementia experience is possible if embedded in the primary framework which in this case is a negative framing of dementia (see Goffman, 1974). By employing conversational practices for constructing alternative frameworks, a potential change in the primary negative framework of the dementia experience may even be possible in the future.

The talk by the couple in this study is collaborative and co-constructed, as the spouses express alignment in their shared talk and affiliation in their assessments, and continuously add to and develop each other’s communicative contributions. This co-constructive element of managing life with dementia fits with previous research which emphasises couplehood rather than individuality (Bielsten et al., 2018; Hellström & Torres, 2021; McGovern, 2011; Swall et al., 2020). Often, the parties’ contributions elicit new information or a possibility to see the described situation from a different perspective. Although both spouses claim that there are occasions when the wife’s abilities fall short, which is in line with the primary negative social framing of dementia (Goffman, 1974), they emphasise the positive assessments of the progression of the dementia. Mathias recurrently accounts for Kari’s shortcomings, for instance, in Ex.#2 when he normalises her difficulties by suggesting that he himself has trouble, and thereby also gets across how difficult baking is. This is also seen in Ex.#3, when Mathias provides an elaborated justifying account of disturbing elements affecting his wife when preparing a meal. Mathias also mitigates the importance of the troubles his wife expresses, for instance in Ex.#1b where he questions whether his wife actually does bake that much anymore. By reducing the impact of the wife’s shortcomings in daily chores as well as providing reasons for them, it is still possible for the wife to preserve a positive face (Brown & Levinson, 1978; Goffman, 1967), as well as to get relevant information across to the interviewers in a storytelling manner.

The couple is successful in terms of making use of their shared experiences and relationship when telling of their experiences of daily life with dementia. For instance, the husband’s recurrent self-deprecations and normalisations in regard to his wife’s abilities work as praise on her behalf and open a possibility for Kari to self-praise and provide elaborated stories about her own capabilities without losing face. A spouse is a third-party ‘expert witness’, and Mathias’ praise is more socially acceptable than for Kari to solely praise herself (Speer, 2011). Being part of an intimate relationship also comes with certain liberties in terms of entering another person’s epistemic domain, specifically in regard to core knowledge such as identity, feelings and hobbies (Bristol & Rossano, 2020). Mathias practices such liberty in Ex.#1b when he suggests that baking is not so much his wife’s hobby anymore. His contribution here is not treated as problematic by his wife, but rather functions as a continuer for Kari to elaborate on her baking story. In this sense, it resembles scaffolding, which has been described as a technique to facilitate further talk from and support persons with dementia (see e.g. Hydén, 2018). Similarly, this study presents instances when epistemic trespassing (i.e. entering another person’s epistemic domain) may function as a supporting practice for encouraging further talk when employed in intimate relationships.

The status of being in an intimate relationship also poses questions regarding epistemics and individuality. Is it actually true that Mathias is entering Kari’s domain when talking of her baking habits, or is it rather that it is a domain which is shared by them since they live together? It has been suggested that the identities of spouses in couples are interdependent (Gottman, 2011), and the boundaries of domains may therefore be more blurred than is suggested in literature on epistemics (e.g. Heritage, 2012). A growing body of literature on spousal relationships and dementia suggests that their couplehood may be superior to their individuality, as their daily life to a great extent is shared with each other (see Nilsson, 2018). In light of that, the trespassing may perhaps not be understood as trespassing but rather part of the recounting of shared experiences. This links back to the issue of praise and self-praise, and raises a question concerning the individuality of praise regarding one person’s contribution to how a couple cope with daily life with dementia. As a couple, this talk may also show them to be like any other (heteronormative) couple in terms of daily chores and responsibilities (see Flinkfeldt, 2014). It may also show them as not being particularly affected by the dementia. So, maybe the praise and self-praise should not be understood as a matter of individuality in this talk, since their identities as an intimate couple may be intertwined (see Bielsten et al., 2018) it can also be understood as a way of praising the couple. This could also explain Mathias’ active involvement in the co-construction of a positive ‘social framing’ (Goffman, 1974) of the dementia experience, as the dementia is not only a matter for his wife but for their shared identity as a couple.

The analysis in this paper puts dementia-specific features on display. For instance, throughout all the interviews there was an underlying expectation that dementia should affect Kari’s capabilities as well as the couple’s daily life and well-being. The expected asymmetry between the spouses in terms of capabilities is made visible in the talk, often embedded in praise for not failing. The couple shows that Mathias is expected to be able to do more things than Kari, and that her capabilities are expected to go downhill. Interestingly, this underlying agreement and presupposition for the talk works as a stepping stone for the ‘positive framing’ on the matter, as it can be contrasted with this worst-case scenario. The dementia aspect also plays an important role in regard to epistemic domains and trespassing into another’s domain. In previous studies, it has been argued that entering the epistemic domain of a person with dementia might be less of a violation than doing the same with a person with intact cognition (Bristal & Rossano, 2020).

Previous research (Ekström et al., forthcoming; Nilsson et al., 2018) has shown how spouses of a person with dementia talk about the challenging times due to their spouses’ dementia in their presence, and through different conversational practices balance the sensitivity and preservation of a positive face in the conversation (Brown & Levinson, 1978; Goffman, 1967) with different means. This study adds to this finding by visualising how the balancing of positive and negative assessments contributes to saving face and minimising the negative effects of dementia on self-worth for Kari. In both spouses’ contributions, there are fine-tuned openings for self-praise for Kari. These conversational practices regarding talk about potentially sensitive information may surface when the talk is directed at a third party, as in an interview context or any other multi-party interaction. Talking about dementia-related issues to a third party may be a delicate matter, and the management of these matters has been addressed in this current study. By looking at examples of conversational practices by a spouse which enhance self-worth and possibilities for self-praise for people living with dementia, we can contribute to a strength-based approach to dementia care, which challenges the deficit model and its focus on loss that is so widely accepted (McGovern, 2011).

The results from this study may inform different institutional contexts such as medical interactions or social work encounters. Previous research on care management assessment has shown how partners of persons with dementia may be more heard than the person with dementia applying for support (Österholm & Samuelsson, 2015). The results from this study suggest more careful listening and awareness of the different parties in couples as their descriptions of the same matter may differ. Although the couple in this study aligned with each other’s communicative turns as well as, specifically in interview two and three, affiliated with the content there may be situations where one of the spouse’s views and assessments receives more weight than the other’s. Disentangling the different parties’ contributions may visualise asymmetries and power relations. In relation to interactions regarding support from social services or medical help, the consequences may be that one person mitigates another person’s subjective descriptions of their shared situation. The shared frame that is presented to others may then be influenced too much by one party’s positive or negative framing, regardless of whether it is the frame of the person with or without dementia. With that said, the conversational practices presented here may also be beneficial for enabling more positive framing or at least alternatives which may challenge or potentially transform the overarching negative framing of life with dementia, which in turn could have positive effects on well-being for those affected.

### Limitations

This is a case study which includes only one couple; it would be beneficial to include more couples who are interviewed over several years to find patterns of conversational practices and sequential organisation for positive framing. Another limitation concerns the context of the interaction. As the interactions in this study were research interviews, the talk between the spouses ought to be understood as performative and aimed at the interviewers as recipients. Hence, no conclusions can be drawn in regard to how the spouses talk behind closed doors. However, couples living with dementia are potentially involved in situations which are similar to this interview context, in that they jointly talk about their life in front of a third party, for instance in medical interactions, meetings with social services or with friends and relatives. Therefore, the results are useful for a wide range of institutional practices which involves couples living with dementia.

## Notes

1 ‘Ju’ is a particle in Swedish which indicates shared knowledge (see Heinemann et al., 2011)

2 Adapted to an English version of the Swedish cooking measurements.

## Appendix

### Transcription key

hh: In-breath
°°: Quieter than surrounding speech
Capital letters: Louder than surrounding speech
< >: Slower than surrounding speech
> <: Faster than surrounding speech
( ): Unheard or unclear utterance
[ ]: Overlapping speech
(.): Pause in seconds
$: Smiley voice
=: No discernible silence between utterances
Prolonged speech
↑ / ↓: Rising/falling intonation
(( )): Non-verbal action
